# Crystal Structure and Conformational Dynamics of N─N Bond‐Forming Piperazate Synthase

**DOI:** 10.1002/cbic.70430

**Published:** 2026-06-17

**Authors:** Nikita Pal, Simon Schröder, Amit Singh Sahrawat, Christian C. Gruber, Martin A. Hayes, Bastian Daniel, Sandy Schmidt, Karl Gruber

**Affiliations:** ^1^ Institute of Molecular Biosciences University of Graz Graz Austria; ^2^ Department of Chemical and Pharmaceutical Biology Groningen Research Institute of Pharmacy, University of Groningen Groningen Netherlands; ^3^ BioTechMed‐Graz Graz Austria; ^4^ Innophore GmbH Graz Austria; ^5^ Field of Excellence BioHealth University of Graz Graz Austria; ^6^ Discovery Sciences BioPharmaR&D AstraZeneca Mölndal Sweden; ^7^ School of Chemistry and Molecular Biosciences University of Queensland St Lucia Australia

**Keywords:** crystallography, heme, MD simulations, N─N bond formation, *N* ^5^‐hydroxy‐ʟ‐ornithine, piperazate synthase

## Abstract

l‐Piperazic acid (l‐Piz) is a noncanonical, *α*‐hydrazino acid characterized by a 1,2‐diazinane heterocycle containing an N─N bond. It occurs in numerous natural products with potent biological activities and represents a key pharmaceutical building block. In nature, l‐Piz is biosynthesized from l‐ornithine via the intermediate *N* ^5^‐hydroxy‐l‐ornithine in a two‐enzyme cascade comprising a flavin adenine dinucleotide (FAD)‐dependent *N*‐hydroxylating monooxygenase (NMO) and a heme‐dependent piperazate synthase (PZS). The NMO selectively hydroxylates the *δ*‐amino group of l‐ornithine, while PZS catalyzes intramolecular N─N bond formation to generate the six‐membered cyclic hydrazine scaffold of l‐Piz. Here, we report the crystal structure, Piz‐forming activity, and molecular dynamics (MD) analysis of SbPZS, a representative PZS from *Streptomyces* sp. B93. High‐resolution structural analysis enabled a detailed comparison with previously characterized PZS homologs. To further delineate the molecular basis of catalysis, we performed MD simulations in combination with sequence‐based bioinformatic analyses. These studies provide insight into protein–substrate interactions, conformational dynamics, and the residues that contribute to active‐site organization. Moreover, we identify candidate hotspots for engineering to modulate substrate scope and catalytic efficiency. Collectively, our results establish a structural framework for understanding enzymatic N─N bond formation in Piz biosynthesis and lay the groundwork for future biocatalytic applications of PZSs.

## Graphical abstract

Piperazate synthases (PZSs) catalyze the key N─N bond formation in the biosynthesis of the rare amino acid piperazic acid. X‐ray crystal structures of apo‐ and holo‐SbPZS show a dimeric enzyme with two heme‐containing active sites, where histidine residues coordinate the iron centers. Structural analysis and molecular dynamics simulations provide insights into cofactor binding, active‐site architecture, and enzymatic N─N bond formation.

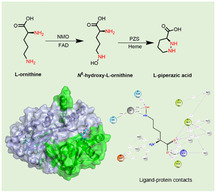

## 1 Introduction

**FIGURE 1:**
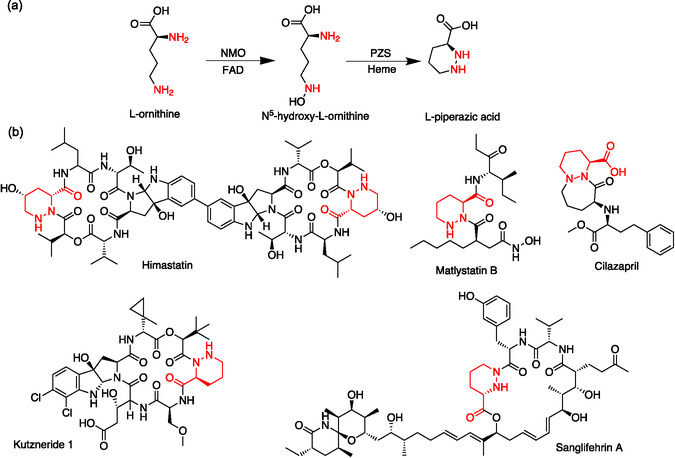
(a) Biosynthesis of l‐piperazic acid. An *N*‐hydroxylating monooxygenase (NMO) hydroxylates the *δ*‐amino group of l‐ornithine, yielding *N* ^5^‐hydroxy‐l‐ornithine, which is further converted to l‐piperazic acid by a piperazate synthase (PZS); (b) l‐Piz containing natural products.

The enzymatic formation of nitrogen–nitrogen (N─N) bonds is a rare and chemically challenging transformation in biological systems. Among the few known examples of N─N bond‐containing compounds, the biosynthesis of l‐piperazic acid ((*S*)‐hexahydropyridazine‐3‐carboxylic acid, l‐Piz) and the identification of the first l‐Piz‐producing gene cluster in *Kutzneria* sp. were accomplished by Walsh and colleagues [1, 2]. Piz is the only naturally occurring amino acid with an N─N bond, first discovered in 1971 as a component of the antibiotic monamycins [3]. It is synthesized via nonribosomal peptide synthesis and mimics the cyclic structure of proline, providing conformational rigidity to these compounds [4]. Compounds containing the six‐membered l‐Piz include Kutznerides (antifungals) [5], sanglifehrins (immunosuppressants) [6], matylstatins (type IV collagenase inhibitors) [7], himastatin (antitumor) [8], and cilazapril (an ACE inhibitor) (Figure 1). In some Kutznerides, the l‐Piz core ring is hydroxylated or chlorinated.

Piperazic acid is formed from l‐ornithine through a two‐step enzymatic process. First, a flavin‐dependent *N*‐hydroxylating monooxygenase (NMO) selectively hydroxylates the *δ*‐amino group of l‐ornithine to produce *N* ^5^‐hydroxy‐l‐ornithine [9]. In the second, and chemically more unusual, step, a heme‐dependent piperazate synthase (PZS) catalyzes an intramolecular cyclization with simultaneous dehydration to form the N─N bond, thereby generating the cyclic hydrazine scaffold of l‐Piz (Figure 1) [10, 11]. Despite significant recent progress, the structural and mechanistic factors that enable this transformation remain incompletely understood.

Through the efforts of Setser et al. [9] and Yi‐Ling Du et al. [10], the initial pathway in the biosynthesis of l‐Piz was characterized. Subsequent studies by Hu and coworkers [12] established the presence and functional relevance of PZS enzymes across several *Streptomyces* strains. More recently, mechanistic insights have emerged from structural, spectroscopic, and computational investigations. In 2025, Higgins et al. [13] proposed a reaction mechanism for this synthesis using electron paramagnetic resonance and QM/MM simulations. The heme‐dependent PZS catalyzes the cyclization, forming an N─N bond between the *α*‐N and *δ*‐N with the removal of water. Complementary work by Yang et al. [14] also proposed a mechanistic model for l‐Piz synthesis by KtzT, in which product formation is catalyzed by the formation of an intramolecular hydrogen bond between the amino and hydroxyl groups of the substrate, leading to dehydration of the hydroxylamine. Furthermore, a water molecule facilitates the formation of a hydrogen bond, thereby bringing the two substrate nitrogen atoms closer together to enable product formation. Another significant discovery regarding PZS was the determination of the crystal structure of LnzB, also reported in 2025 [15]. These studies highlight the importance of precise substrate conformations and underscore the need for high‐resolution structural information to define how the protein scaffold permits such reactive conformations.

The substrate specificity of this class of enzymes has recently been explored across a broader range of substrates, including multiple diamino acids and diamine derivatives [16]. Moreover, the presence of a carboxylic group in the substrate is not required, and the enzymes exhibit varying enantiomeric preferences. Among the studied PZSs, KtzT was the most promiscuous enzyme. These findings raise the possibility of engineering PZSs for the synthesis of noncanonical hydrazine‐containing building blocks.

In this study, we report the structural and computational characterization of a PZS from *Streptomyces* sp. B93 (SbPZS), which shares 54% sequence identity with KtzT. A His‐tagged SbPZS was recombinantly overproduced in *E. coli* and purified. After reconstitution with heme, we determined the crystal structure of the holoenzyme at 2.1 Å resolution. Heme imparts a prominent reddish‐brown color to the protein solution and crystals, and produces a Soret peak at 418 nm, confirming the presence of the cofactor‐bound protein. Comparative structural analysis with previously reported PZS enzymes reveals a highly conserved overall architecture while highlighting differences. To further investigate protein–substrate interactions and substrate dynamics, we performed molecular dynamics (MD) simulations of SbPZS in complex with *N* ^5^‐hydroxy‐l‐ornithine, complemented by bioinformatics analysis of conserved active‐site residues across a broad set of homologs. Together, these results provide a structural framework for understanding N─N bond formation in PZSs and identify potential hotspots for future enzyme engineering.

## 2 Results and Discussion

### 2.1 Purification and Oligomeric State Determination

SbPZS was expressed in *E. coli* NiCo21 (DE3) cells, purified by affinity and size exclusion chromatography (SEC), and concentrated to 10 mg/mL. SEC–MALS results indicate that the molecular weight of the protein is 36.2 kDa, while the expected molecular weight of an SbPZS dimer with hexa‐His‐Tag is ≈50.8 kDa. The discrepancy between the observed and expected molecular weights can be attributed to variations in the dn/dc values used for data analysis of hemoproteins (Supporting Information (SI) Figure S1).

### 2.2 Cofactor Loading/Heme Reconstitution

As the purified protein had a low heme content, we performed heme reconstitution. Testing various hemin (chloride) concentrations with the protein using UV–Vis spectroscopy revealed a Soret peak at ≈418 nm, indicating heme binding. The absorbance maxima at ≈418 nm can be explained by the presence of a His‐Tag in the protein, as also observed by Higgins et al. [13]. We found that a twofold excess of hemin is sufficient to saturate the protein solution (SI Figure S2). Any additional heme beyond this point results in a significant increase in nonspecific interactions.

Evaluation of the thermostability of SbPZS before and after reconstitution showed that the melting temperature increased from 46°C to 56°C, indicating enhanced protein stability upon cofactor loading (SI Figure S3).

### 2.3 Piperazate Synthase Activity

N─N bond formation leading to l‐Piz was confirmed by liquid chromatography–mass spectrometry (LC–MS) analysis of reactions containing heme‐reconstituted SbPZS and *N* ^5^‐hydroxy‐l‐ornithine. A product peak corresponding to l‐Piz (*m*/*z* 131.0 [M + H]+, retention time 2.1 min) was detected and matched the authentic standard (SI Figure S4). No product was observed in the no‐substrate control, confirming that product formation depends on the complete enzymatic reaction mixture. These data provide experimental support for the assignment of SbPZS as a functional piperazate synthase.

### 2.4 Structure Determination

**FIGURE 2:**
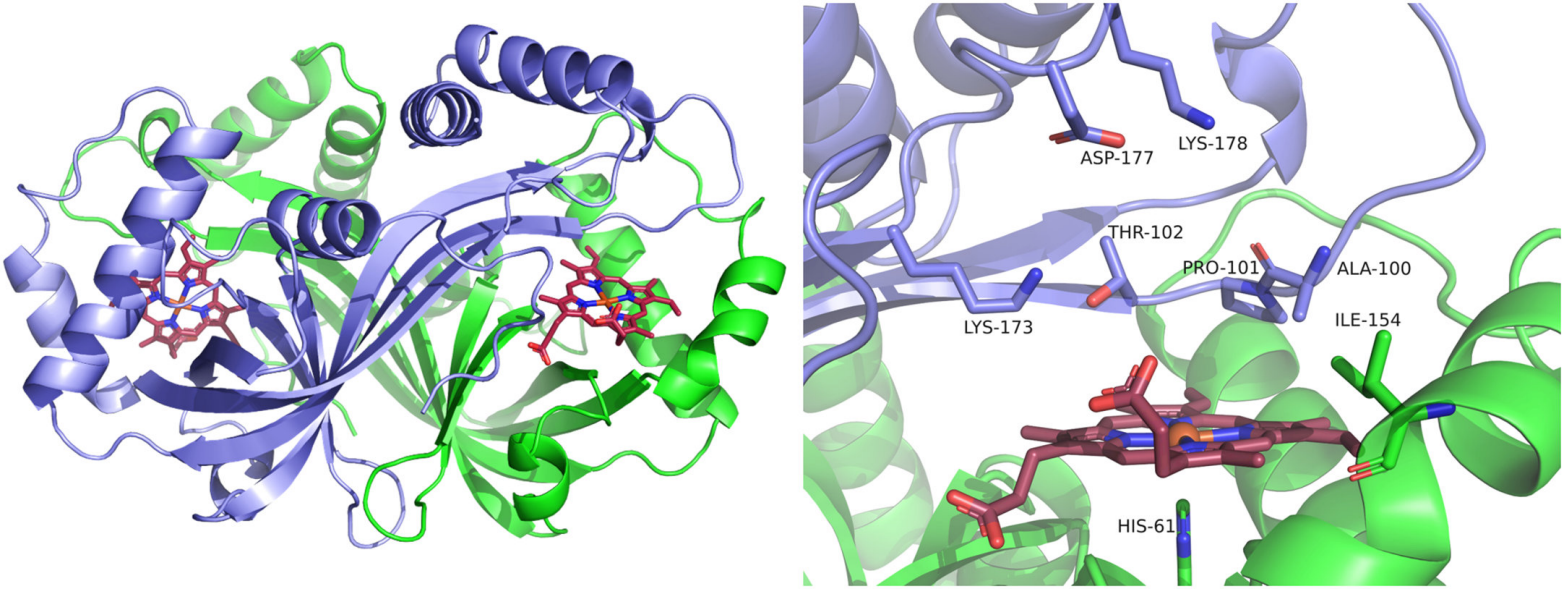
Left: Cartoon depiction of the SbPZS dimer with heme bound in the active sites, with chains of the homodimer colored in blue and green. Right: The active site of SbPZS with heme, showing the folded conformation of the heme’s propionic acid side chain. The 3D visualization of this structure can be found here: (https://nikitapal9.github.io/PZS_3D_model/) [17].

Initial crystals of SbPZS were obtained from purified protein samples before heme reconstitution. These tetragonal crystals (space group P 4_3_2_1_2) diffracted to 2.3 Å resolution, and the lack of significant electron density for the heme indicated very low cofactor loading, consistent with the spectroscopic analyses. These apo‐SbPZS crystals were then used as microseeding nuclei to successfully generate holo‐SbPZS crystals. Diffraction data for holo‐SbPZS were extended to a resolution of 2.1 Å with trigonal symmetry (P 3_2_) (SI Table S1). Structural analysis showed that SbPZS (PDB: 9QLK) adopts a homodimeric architecture (Figure 2) and has an approximate molecular weight of 46.8 kDa, consistent with other homologs (PipS, KtzTC197S, and LnzB). Within the asymmetric units, six SbPZS molecules pair to form three dimers, arranged via a noncrystallographic twofold axis. These dimers are structurally highly conserved (RMSD values up to 0.27), especially in regions critical for enzymatic activity. Heme conformation, the spatial arrangement of catalytic amino acids, and the placement of water molecules involved in hydrogen bonding. Each monomeric unit features a split *β*‐barrel at the dimer interface with its counterpart in an antiparallel fashion, flanked by *α*‐helices.

The two active sites are located at the dimer interface and are formed by residues from both chains (Figure 2). A heme moiety is positioned within this cavity (SI Figure S5), with the iron coordinated by His‐61 at a distance of 2.1 Å (SI Figure S6). One heme propionate adopts a conformation compatible with substrate interaction, whereas the second extends into the cavity and contacts Thr‐38, the main‐chain NH group of Asn‐63, and the side‐chain carboxamide of Asn‐66. Additional electron density above the heme is observed in chains A–E but not in chain F; this density is compatible with a histidine residue, most likely from the C‐terminal hexa‐His tag (SI Figure S7). Because no continuous density connects this residue to a specific chain, its chain of origin remains uncertain.

### 2.5 Structural Comparison of Piperazate Synthase Homologs

**FIGURE 3:**
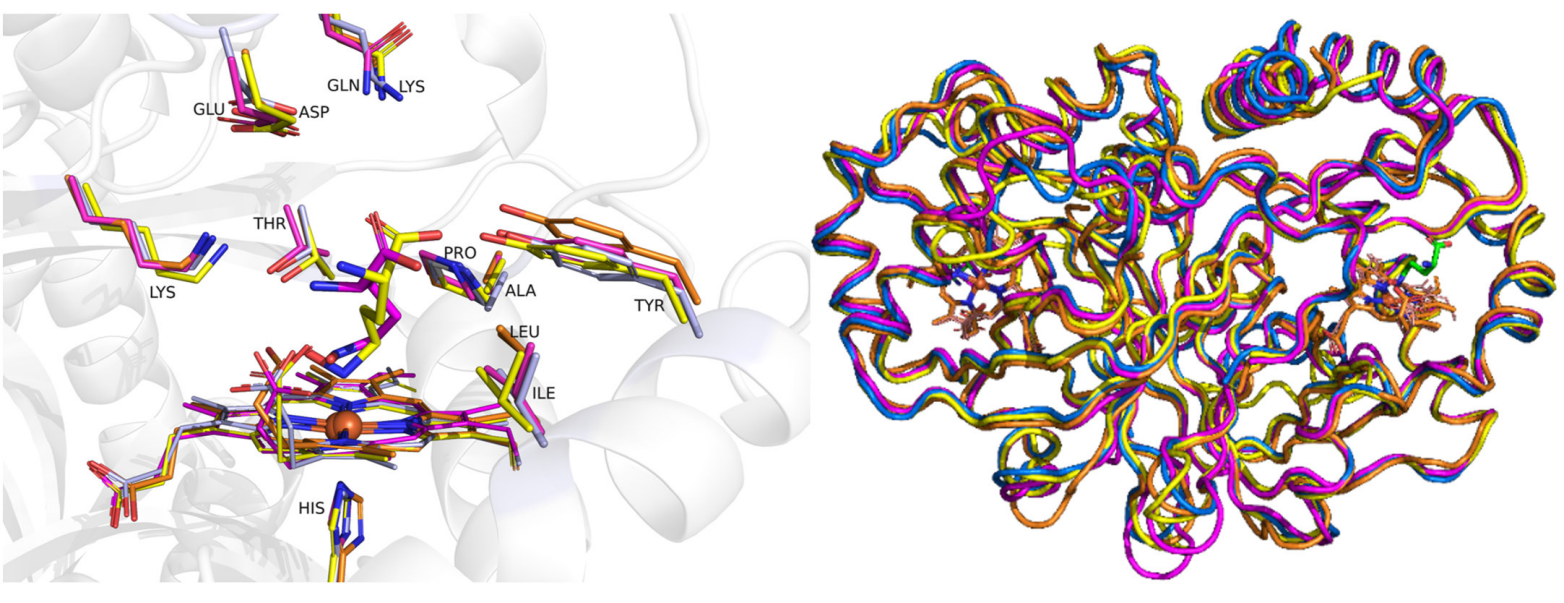
Left: Stick representation of significant amino acid residues in the active sites of SbPZS in light blue (9QLK), PipS in yellow (9EBM), KtzT in pink (9JN5), and LnzB in orange (9KEA). Right: Ribbon representation of crystal structures superimposed, SbPZS (blue), PipS (yellow), KtzT (pink), LnzB (orange).

The crystal structures of all PZSs, including PipS (9EBM), KtzT (9JN5), and LnzB (9KEA) available to date, are highly similar to each other (Figure 3) with similar overall shape and folds, including ≈340 aligned Cα atoms, and show low RMSD values between 0.5 and 0.9 Å (SI Table S2). The orientation of the *α*‐helices and *β*‐strands is highly similar, while the loops show some variability. Consistent with previous studies, His‐61 is positioned for heme coordination, and the polarity and geometry of the active‐site cavity are conserved. Thr‐102, Tyr‐150, Lys‐173, and Lys‐178 correspond to residues implicated in catalysis in other PZS homologs [13, 14]. Some residues in the active site exhibit slight variability, yet they maintain a similar environment with respect to charge, polarity, and hydrophobicity. Additionally, cofactor binding is identical among the three in orientation and interactions with the protein. For protein–substrate interactions, the substrate conformations observed in PipS and KtzT are fairly distinguishable. A conformational change in the substrate with a 90° rotation around the C1‐N5 axis was observed in the crystal structure of KtzT [14], enabling interaction with the heme’s propionic acid side chain. During this change in conformation, the distance between the two nitrogen atoms decreases from 4.9 Å (extended) to 3.7 Å (closed), making the substrate more susceptible to N─N bond formation. By contrast, in the crystal structure of PipS, the substrate is presented in an extended conformation where the *α*‐amino group interacts with the propionic acid side chain of heme (3.0 Å distance) and the *α*‐carboxy group interacts with the Y155 of PipS. The distance between the two nitrogen molecules in this case is between 5.1 and 5.2 Å. We hypothesize that the starting substrate conformation in SbPZS is analogous to that observed for PipS. This hypothesis is supported by an AlphaFold‐predicted model, which shows a comparable substrate orientation within the active‐site framework. Consistent with this, our MD simulations reveal that the overall substrate RMSD remains low over the trajectory, indicating that the predicted conformation is maintained under these conditions (SI Figure S12).

A tunnel aligned with the N‐terminus of SbPZS may facilitate water transport to the active site or serve as a proton relay (SI Figure S8). Water molecules within this tunnel are highly conserved across all three crystallographically independent dimers, except for additional water molecules observed in dimer 1. This tunnel appears too narrow for substrate entry, whereas the space above the heme is solvent accessible, providing a plausible access route for the substrate (SI Figure S9). In several modeled PZS homologs, however, N‐terminal truncation creates additional space near the active site, suggesting that this region may influence water or substrate access.

Intriguingly, PZSs share structural similarity with PaiB [18], a transcriptional regulator originally not associated with N─N bond formation. On further comparison with PaiB, the overall protein fold remains well conserved (with a Cα RMSD of 1.6 Å over 279 aligned residues). Still, some *α*‐helices and *β*‐sheets are shorter and contribute to disordered loop regions. The N‐terminus of PaiB also lacks certain residues relative to the PZS. Although PaiB was shown to bind heme (PDB ID: 9VYC) and contains residues important for binding and catalysis (His‐61, Asn‐66, Thr‐102, and Lys‐173) in PZS, it lacks N─N bond‐forming catalytic activity (SI Figure S10). The similarities between PaiB‐like proteins and *bona fide* PZSs indicate an evolutionary relationship [15]. Due to these few similarities, most PZSs are annotated as FMN‐binding proteins.

### 2.6 Bioinformatic Analysis

**FIGURE 4:**
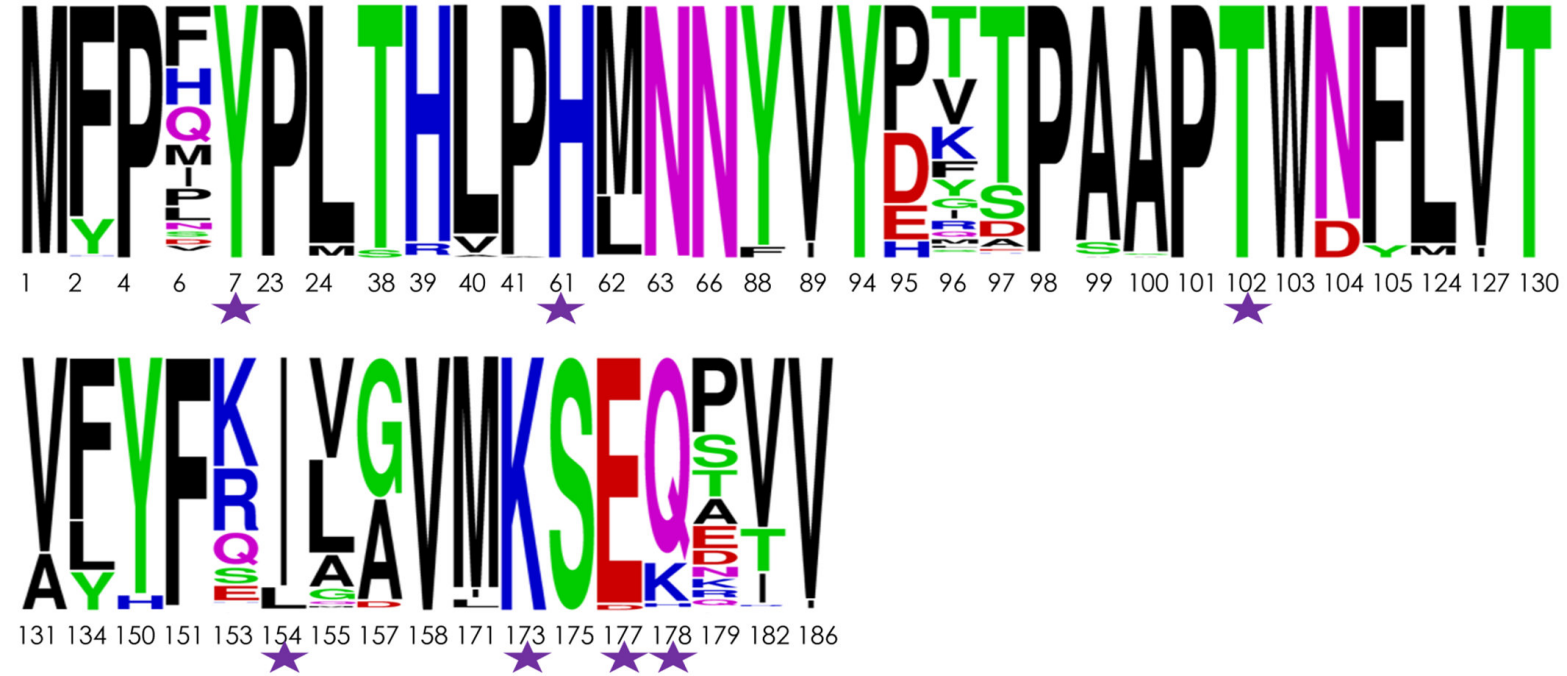
A sequence logo generated between 478 homologs to evaluate the residues around the active site of PZSs. The height of the letters signifies their conservation levels. Color code: Green for polar amino acids, blue for positively charged amino acids, red for negatively charged amino acids, and black for hydrophobic amino acids. All known significant amino acids are marked with a star. The sequence logo was created using WebLogo [20].

A search of the NCBI database for SbPZS homologs revealed that this protein family is highly conserved across species. Notably, even among the first 1000 BLAST hits [19], sequences still share up to ≈45% identity with the query, highlighting the strong evolutionary conservation of this class of enzymes. To further investigate conservation patterns within functional regions, we analyzed the active site and protein–substrate interactions using sequence logo representations (Figure 4). This analysis provided important insights into residue variability across the protein. Greater sequence divergence was observed primarily in surface‐exposed residues such as Val‐6, Ser‐96, Lys‐153, and Glu‐179, whereas residues located near the substrate and cofactor remained strongly conserved. Residues including Tyr‐7, His‐65, Thr‐102, Ile‐154, Lys‐173, Asp‐177, and Lys‐178, which are known to be catalytically significant [13, 14, 21], are well conserved. These findings suggest that while peripheral regions of the protein may tolerate substitutions, the catalytic core is under strong selection to maintain the piperazate synthase activity. The observed variation patterns, therefore, offer valuable guidance for identifying potential engineering targets, particularly in regions adjacent to the active site, where modification may be feasible without compromising enzymatic activity.

### 2.7 Molecular Dynamics Simulations

The proposed catalytic mechanism proceeds via a heme iron center that supports protein–substrate interactions. Iron plays an active role in catalyzing the reaction through coordination and electron redistribution. In particular, binding to the heme enables activation of the –NH–OH, formation of Fe–N species, promotes hydroxylamine functionalization, and subsequent rearrangements that lead to cyclization. Importantly, the outcome of this activation is strongly influenced by the conformation of the substrate within the active site. We postulate that cyclization to the hydrazine product necessitates close spatial proximity between the two nitrogen atoms of the substrate. To evaluate this requirement, we monitored the intramolecular nitrogen distance throughout the simulations as a conformational descriptor (SI Figure S11). The analysis reveals that the closed conformation, defined by an N─N separation of less than 4 Å, is a rare event as the substrate rapidly reverts to an extended geometry with the two nitrogens separated by more than 4 Å and up to 6 Å distance. Given that the two active sites of a dimer are functionally independent, stochastic variations were observed between them; thus, they can be regarded as two statistically independent simulation systems.

Because the heme propionate can interact with the substrate, we compared the conformational stability of this side chain in simulations with and without substrate. The two monitored dihedral angles (SI Figure S13) sampled two main conformational clusters centered approximately at (−120°, 60°) and (−80°, −60°), indicating that the propionate can interconvert between two preferred states. In substrate‐bound simulations, the distribution was more concentrated around the major cluster, consistent with stabilization of the propionate conformation by substrate contacts. In substrate‐free simulations, broader sampling indicated greater propionate flexibility.

**FIGURE 5:**
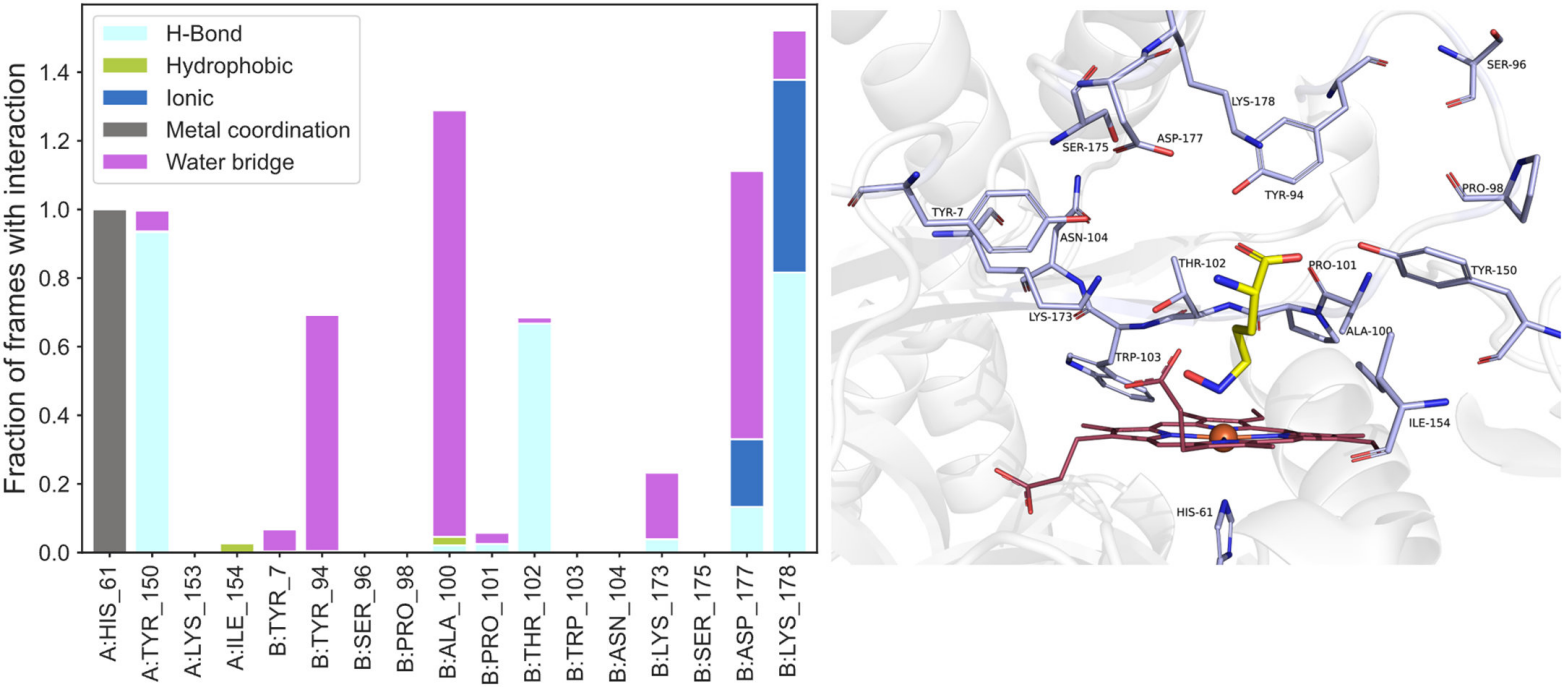
Left: Protein–ligand interactions are categorized into four types: H‐bonds, hydrophobic, ionic, and water bridges. The stacked bar charts are normalized throughout the trajectory; a value of 0.7 indicates that 70% of the simulation time is spent on the specific interaction. Values greater than 1.0 are possible, as some protein residues may form multiple contacts of the same subtype with the ligand. (Schrödinger 2025–3). Right: Active site of SbPZS highlighting the key residues involved in protein–ligand interactions. A 3D visualization of this structure is available here: https://nikitapal9.github.io/active_site_aa/ [17]. The critical residues are shown in a stick representation.

Protein interactions with the ligand are monitored during the simulation, classified by type, and summarized (Figure 5) to assess the influence of key residues on ligand binding and protein structural stability. Analysis of these interactions shows that His‐61 consistently forms a coordination bond with the heme iron, indicated by a value of 1.0, highlighting its essential role in anchoring the catalytic cofactor. Additionally, Tyr‐94, Ala‐100, Tyr‐150, Pro‐101, Asp‐177, and Lys‐178 interact with the substrate via hydrogen bonds and water bridges. Among these, Asp‐177 and Lys‐178 exhibit the strongest interactions, mediated by multiple points of contact, suggesting that they play a central role in stabilizing the bound substrate. Ala‐100 and Ile‐154 form hydrophobic interactions with the substrate. Collectively, these residues represent potential targets for mutation or engineering studies on SbPZS to investigate substrate promiscuity. Some of these residues, such as Thr‐102 and Lys‐173, play a significant role in catalysis and are known to influence l‐Piz production [13, 14, 21].

## 3 Conclusion

This study offers biochemical, structural, and computational insights into SbPZS, a heme‐dependent enzyme that catalyzes N─N bond formation during l‐Piz biosynthesis. With the increasing demand for pharmaceutically relevant compounds, enzymes involved in the production of l‐Piz and other cyclic hydrazines are becoming more valuable as potential biocatalysts. This work aimed to clarify the structural framework underlying enzymatic N─N bond formation by integrating MD simulations with existing mechanistic knowledge of the PZS enzyme family, including PipS, KtzT, and LnzB. Structural comparison of SbPZS with other characterized PZSs revealed a highly conserved catalytic architecture, with some variations that may contribute to functional specificity.

Our results support the conclusion that PZSs depend on both the heme cofactor and the precise conformation of *N* ^5^‐hydroxy‐l‐ornithine to enable productive catalysis. MD simulations further show that ligand binding is stabilized by an organized network of interactions, including persistent heme coordination by His‐61 and strong electrostatic contacts mediated by residues such as Asp‐177 and Lys‐178. Once coordinated, the iron center begins to polarize the N─O bond of the hydroxylamine. Together, these findings highlight the central importance of substrate positioning and active‐site dynamics in enabling efficient N─N bond formation. The formation of the N─N bond is a chemically complex transformation that requires differentiation of the two nitrogen atoms into electrophilic and nucleophilic species [13]. Consistent with the mechanisms proposed for PipS and LnzB, key residues, including Thr‐102 and Lys‐173, facilitate hydroxyl‐group elimination to generate a reactive nitrogen species by hydrogen bonding with the ─NH─OH group and promoting water elimination during cyclization. However, in KtzT, the interactions between the substrate and Thr‐Lys dyad are mediated by a solvent‐derived water molecule, suggesting mechanistic differences among the PZS homologs.

The oxidation state of the heme iron in PZS catalysis remains unresolved. Current evidence suggests that in vitro Piz formation may be initiated from the Fe(III) state of the heme [13], though further studies are needed to define the catalytic redox cycle. The heme propionate side chain is also likely to be mechanistically important through hydrogen bonding to the *α*‐amino group, as suggested by previous studies [21] and observed in our MD simulations. In our simulations, the zwitterionic substrate form maintained productive contacts with both the protein and the cofactor, consistent with recent computational models [13, 14]. Because the physiological protonation state has not been directly established, we present this as a modeling‐supported interaction pattern rather than definitive evidence for a single substrate form. While the detailed pathway remains incompletely understood, the present work provides a foundation for future mutagenesis and protein engineering studies aimed at exploring substrate promiscuity and improving enzyme stability.

## 4 Experimental Section

### 4.1 Purification

Competent *E. coli* NiCo21 (DE3) cells containing the chaperone plasmid pKJE7 were prepared and transformed with pET28a‐SbPZS (C‐terminal hexa‐His‐tag) (UniParc ID: A0A941BP51) using a standard bacterial transformation procedure. Transformed colonies were grown overnight in LB medium at 37°C and used to inoculate main cultures supplemented with l‐arabinose (500 mg/L), kanamycin (30 mg/L), and chloramphenicol (50 mg/L). At an OD_600_ of 0.4–0.6, cultures were equilibrated at 20°C for 30 min before overexpression was induced with 0.4 mM IPTG for 16–20 h. Cells were harvested, washed, and lysed in 50 mM Tris and 500 mM NaCl (pH 7.0) by sonication (80% amplitude, 20 min half cycles). The soluble fraction was purified by Ni‐NTA affinity chromatography (HisTrap FF 5 mL column, Cytiva) using a 50–500 mM imidazole gradient. Before loading, the sample was filtered with syringe filters (1.0 and 0.45 μm). Further purification was performed by SEC using a Superdex 200 Increase 10/300 GL column (Cytiva) on an Äkta Avant system (Cytiva). UV absorbance at 280 and 418 nm was used to monitor protein and heme‐containing fractions, respectively. The SEC was coupled to multi‐angle light scattering (MALS) to evaluate the oligomeric state of purified SbPZS in solution.

### 4.2 Cofactor Binding

To increase the heme saturation of SbPZS, we tested several methods, including adding hemin during cell lysis and supplementing with ferrous sulfate and 5‐aminolevulinic acid during expression. Ultimately, heme reconstitution was the most effective method for achieving high heme loading. Affinity‐purified protein was incubated overnight at 4°C with heme at concentrations ranging from 0 to 10 molar equivalents relative to the protein to determine the amount required to supersaturate the heme‐binding sites. Excess heme was removed by washing the protein samples with 250 mM imidazole.

### 4.3 Piperazate Synthase Activity

Reaction mixtures containing as‐purified SbPZS (1 µM), hemin chloride (5 µM), and *N* ^5^‐hydroxy‐l‐ornithine (OH‐Orn, 5 mM) in potassium phosphate buffer (50 mM, pH 8.0) were incubated at 25°C for 3 h with continuous shaking. All reactions were performed in triplicate. Subsequently, the reactions were quenched by a 1:100 (*v*/*v*) dilution into acetonitrile/water (78:22, *v*/*v*) containing 0.1% formic acid and analyzed by LC–MS.

LC–MS analysis was carried out on an Acquity Arc UPLC system coupled to an Acquity QDa single‐quadrupole detector (both Waters), equipped with a NUCLEOSHELL HILIC column (150 × 2 mm, 2.7 µm; Macherey‐Nagel) at 40°C. 1.5 µL of the sample was injected onto the column. The mobile phase consisted of water (solvent A) and acetonitrile (solvent B), each supplemented with 0.1% formic acid, at a flow rate of 0.5 mL/min. The following gradient was applied: 0–2 min, 22% A (isocratic); 2–7 min, linear increase to 50% A; 7–9 min, 50% A (isocratic); 9–12 min, return to 22% A for column re‐equilibration. Detection was performed by electrospray ionization in positive mode with a probe temperature of 300°C and a capillary voltage of 1.0 kV (positive)/0.8 kV (negative). Selected ion recording was set to *m*/*z* 131.0 [M + H]^+^ for piperazic acid (Piz; cone voltage 11 V) and *m*/*z* 149.0 [M + H]^+^ for OH‐Orn (cone voltage 8 V). Under these conditions, Piz and OH‐Orn eluted at 2.1 and 7.1 min, respectively. Analyte identities were confirmed by comparison of retention times and *m*/*z* values with those of authentic standards. Piperazic acid hydrochloride, used as a standard, was purchased from BLD Pharm, and OH‐Orn was synthesized according to previously described procedures [10].

### 4.4 Crystallization, Diffraction Analysis, and Structure Determination

The Douglas Instruments Oryx8 protein crystallization robot was used to set up 96‐well plates for sitting‐drop vapor‐diffusion crystallization. The crystallization plates were prepared with 10 mg/mL protein and the JCSG + Eco screen (Molecular Dimensions), incubated at 20°C. Reddish‐brown, diamond‐shaped crystals were obtained in 0.2 M sodium malonate, pH 7.0, containing 20% PEG 3350. Diffraction data were collected on the ID30A‐3 beamline at the European Synchrotron Radiation Facility (ESRF), Grenoble. The initial crystal structure of the SbPZS apoprotein, refined to 2.3 Å resolution, was determined by molecular replacement using an AlphaFold2 [22] model as the search template. No electron density for the heme cofactor was observed, indicating a low level of heme loading in the overexpressed protein as purified. However, the apoprotein crystals were used as seeding nuclei to facilitate crystallization of the heme‐bound holoprotein after in vitro cofactor reconstitution (see section Cofactor Binding). The seed stock was prepared according to the procedure described by D’Arcy et al. [23]. Holoprotein crystals (brown needle‐shaped) were obtained from 0.16 M calcium acetate, 0.08 M sodium cacodylate, pH 6.5, and 14.4% PEG 8000.

Diffraction data were collected on the ID23‐2 beamline at the ESRF, Grenoble (https://doi.org/10.15151/ESRF‐ES‐2210529250) at 100 K from cryocooled crystals after soaking them in 20% glycerol for cryoprotection. The autoprocessed data obtained from the ESRF were processed via GrenADES [24], and the initial apoprotein structure was then used for molecular replacement with Molrep [25]. Clear electron density for the heme cofactor was visible immediately after molecular replacement. Refinement, model building, and addition of water molecules were performed using the software packages Refmac [26], Phenix [27], and Coot [28]. The refined coordinates and structure were deposited in the Protein Data Bank under the accession code 9QLK (pdb_00009qlk). Details of data collection and refinement statistics are available in SI Table S1.

### 4.5 Bioinformatics Analysis

A systematic approach was used to identify and analyze homologous protein structures. A BLAST search identified 478 SbPZS homologs, providing a comprehensive dataset for structural and functional analysis. These homologs were subjected to structure prediction using AlphaFold3 [29], generating high‐confidence models. The predicted structures were aligned using PyMOL (The PyMOL Molecular Graphics System, version 2.5, Schrödinger, LLC) to identify conserved features and structural variations among homologs. Based on this structural alignment, 49 residues within and around the active site were selected, as they are likely crucial for the protein’s function, stability, or interactions, and serve as a foundation for further computational and experimental studies. A sequence logo (Figure 4) was generated using WebLogo [20] from the structural alignment.

### 4.6 Molecular Dynamics Simulations

All MD simulations were performed using the Desmond package [30] in Schrödinger (2025–3) Suite’s Maestro (14.4). The setup included the crystal structure of SbPZS in complex with the substrate *N* ^5^‐hydroxy‐l‐ornithine, which was comodeled using AlphaFold3 [29]. The predicted substrate conformation closely matches that observed in the PipS complex structure (PDB: 9EBM) [13]. The protein was prepared by including the heme cofactor and assigning zero bond order between the metal and the ligand. Protein and ligand preparation workflows were used to check for missing atoms and assign protonation states. The protein was solvated in an orthorhombic box with a 10 Å buffer, and the extended simple point charge (SPC/E) water model was applied with periodic boundary conditions. The solvated protein–ligand system was neutralized with Na^+^ and Cl^−^ ions at a 0.15 M salt concentration. The OPLS5 [31] polarizable force field, specifically designed to improve the modeling of metal–organic complexes, was used to model the potential energy of the protein, ligand, and ions. Equilibration and relaxation were performed in the isothermal–isobaric (NPT) ensemble, maintaining a constant number of particles, a temperature of 300 K (Langevin thermostat), and a pressure of 1.01 bar (Langevin barostat). Trajectories were recorded for 250 ns, with snapshots saved every 10 ps. RMSD plots of the protein and the ligand show proper structure equilibration (SI Figure S12). To ensure statistical reliability, three independent 250 ns simulations were conducted. The results were analyzed with respect to changes in substrate conformation as a function of the intramolecular distance between the two nitrogen atoms of the substrate. Additionally, a comparative analysis of protein–substrate interactions between extended and closed substrate conformations (from the MD trajectories) was performed to observe conformational changes in the active site and protein structural stability. The conformational stability of the heme side chain potentially involved in catalysis was also evaluated with/without the substrate in the active site.

## Funding

This study was supported by European Commission (101073065).

## Conflicts of Interest

The authors declare no conflicts of interest.

## Supporting information

The supplementary material (PDF, 12 pages) includes DNA and protein sequences, 13 supplementary figures, and 2 supplementary tables.

## Data Availability

The data that support the findings of this study are openly available in Protein Data Bank at https://www.rcsb.org/, reference number pdb_00009qlk.
