# Supplementary material for: Crystal Structure and Conformational Dynamics of N─N Bond‐Forming Piperazate Synthase
Source: Chembiochem. 2026 Jun 17;27(12):e70430. doi: 10.1002/cbic.70430 (PMC13274692; doi:10.1002/cbic.70430)
Supplement: Supplementary file 1 — The supplementary material (PDF, 12 pages) includes DNA and protein sequences, 13 supplementary figures, and 2 supplementary tables. [file CBIC-27-e70430-s001.pdf]

Crystal Structure and Conformational Dynamics of N–N Bond-forming Piperazate Synthase - Supplementary Material

Nikita Pal, Simon Schröder, Amit Singh Sahrawat, Christian Gruber, Martin A. Hayes, Bastian Daniel, Sandy Schmidt, and Karl Gruber

1. <u>Sequences</u>

Gene sequence SbPZS-hexa-His-Tag (pET28a)

5’----
ATGGCAATGTATGTTCCAGCTGTTTATCAAGCGCGTGAAGGGCGTCAATTGGTAGAAGTTGTTTCACA
ATATCCATTAGCTGTGTTGATGACAAACGGTCCAAGCACTCCATTCTCGACCCATTTACCTGTAATCC
CGGCTAGCGAGACGGATGTAGATGAGTTGGTAGGATCAACCCTTCTGGGGCACATGAACCGGGCTA
ACCCCCATTGGAGCGCTTTGAGAGCTGGTATAGCTGCGAAAGCAGTATTTTGGGGTCCCAACAGTTA
CGTCACTCCCATGTTATATCCATCAGATCCTGCAGCTCCAACATGGAATTTTGTTTCTGTTCATGTAGA
GGGTGTACTGCAACCTGTTCATGATGATGAAGAAACGCTGGCAGTGGTTCGTCGGACAGCAGCGCG
TTTAGAGGGGCGTTTTGGGGCGGGTTGGGACCAAGAGGGGAGCTTAGATTATTTTCGTAAAATACTG
CCGGGAGTCGGTGCATTTCGACTTGAGGTACGGTCAGCCCAAGGCATGTTTAAACTGTCACAAGATA
AAGAACCGGCAGTTCGTCGACGTATTCGAGAACATTTTGAAGCCGATGGAACTGGTCCTACGCGCGA
GCTGGGTCGTGCTATGAGAAATTTTGATGAGGCAACCGAGAGAGAATATGGTGCGCTCGAGCACCA
CCACCACCACCACTGA ----3’

E. coli NiCo21(DE3) cells used for expression were purchased from New England Biolabs (catalog no. C2529H).

Protein sequence SbPZS (UniParc: A0A941BP51)

*MA*MYVPAVYQAREGRQLVEVVSQYPLAVLMTNGPSTPFSTHLPVIPASETDVDELVGSTLLGHMNRANP
HWSALRAGIAAKAVFWGPNSYVTPMLYPSDPAAPTWNFVSVHVEGVLQPVHDDEETLAVVRRTAARLEG
RFGAGWDQEGSLDYFRKILPGVGAFRLEVRSAQGMFKLSQDKEPAVRRRIREHFEADGTGPTRELGRA
MRNFDEATEREYGA*LEHHHHHH*

The first two amino acids (MA) are cloning artifacts. Residues 3-220 correspond to Uniprot entry A0A941BP51. The last 8 amino acids (LEHHHHHH) constitute a purification tag.

2. Supplementary Figures

Protein purification and oligomeric state determination:

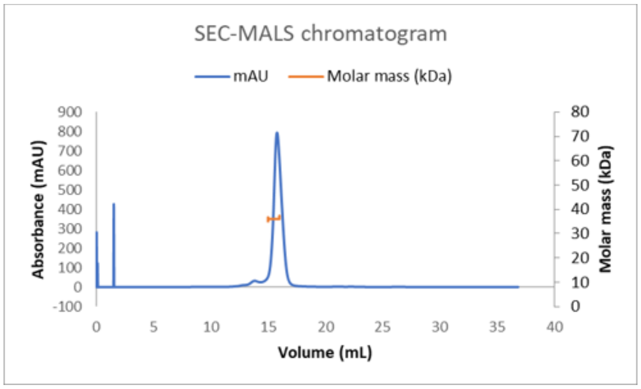

Figure S1: SEC-MALS chromatogram of purified SbPZS. The observed molecular weight was 36.2 kDa, whereas the expected molecular weight of the His-tagged SbPZS dimer is approximately 50.8 kDa. The discrepancy is likely attributable to the dn/dc value used in hemoprotein analysis.

Cofactor loading:

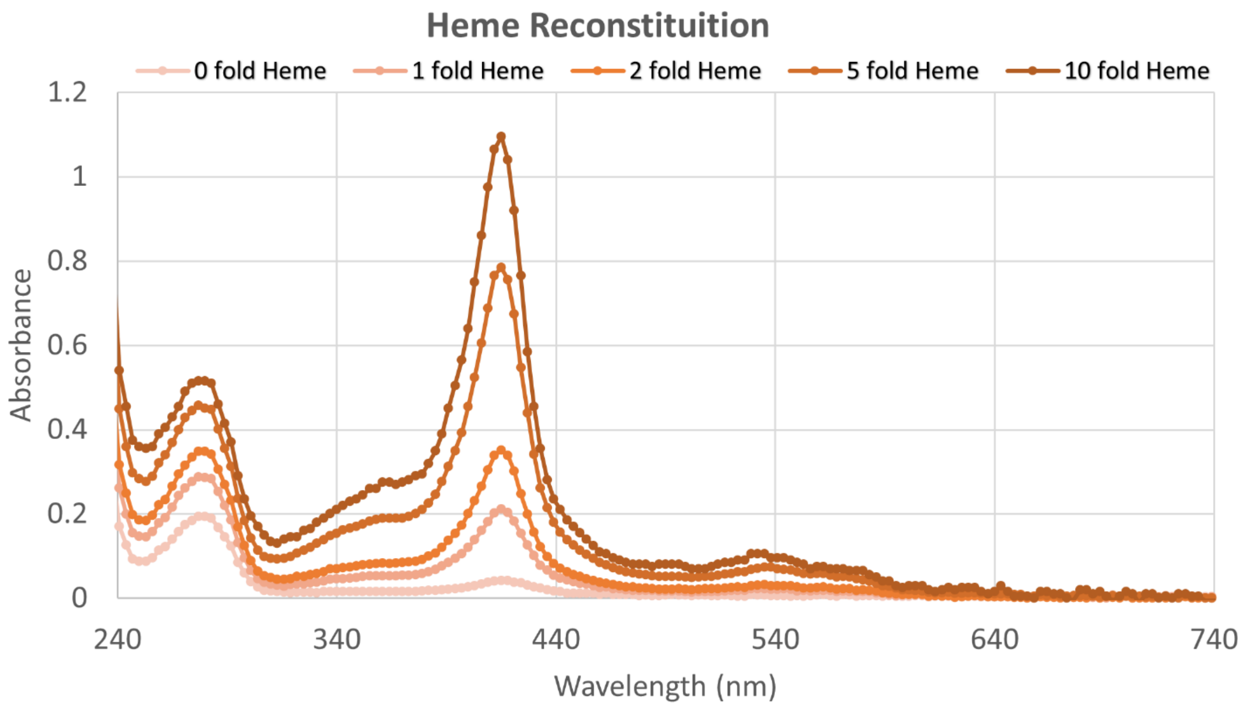

Figure S2: UV-Vis analysis of heme reconstitution of SbPZS. The Soret band near 418 nm indicates heme binding, and saturation was observed at approximately two molar equivalents of hemin relative to protein.

CD temperature scan:

To monitor changes in the secondary structure and thermal stability of SbPZS, a temperature scan was performed using a Jasco J-1500 CD spectrophotometer. Before measurements, the protein sample buffer was exchanged for 100 mM potassium phosphate buffer, pH 7.0, and the purified protein concentration was adjusted to 0.1 mg/mL. An upscan from 20 °C to 95 °C was performed to observe protein denaturation.

Circular dichroism (CD) thermal stability assays were conducted to determine the thermostability of the purified SbPZS protein at 222 nm. The melting temperature (Tm) of the apo-form of SbPZS was 46 ± 0.13 °C. Notably, after heme incorporation, the Tm of the holo-form of SbPZS increased significantly to 56 ± 0.06 °C. The increase in Tm upon heme reconstitution indicates that cofactor binding stabilizes SbPZS under the assay conditions. No significant structural changes in the active site were observed after heme binding.

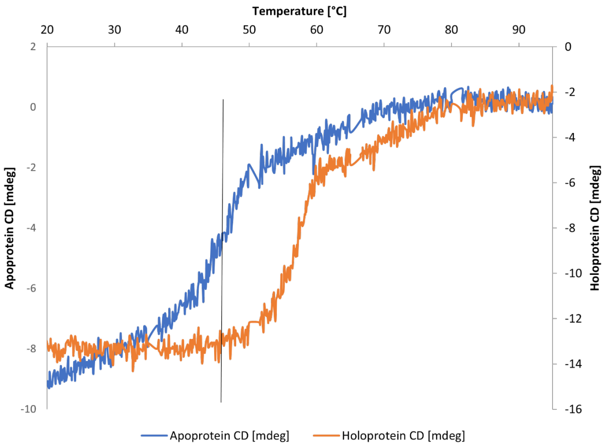

Figure S3: CD thermal denaturation of as-purified SbPZS (blue) and heme-reconstituted SbPZS (orange) monitored at 222 nm. The apparent Tm increased from 46.0 ± 0.13 °C to 56.0 ± 0.06 °C upon heme reconstitution.

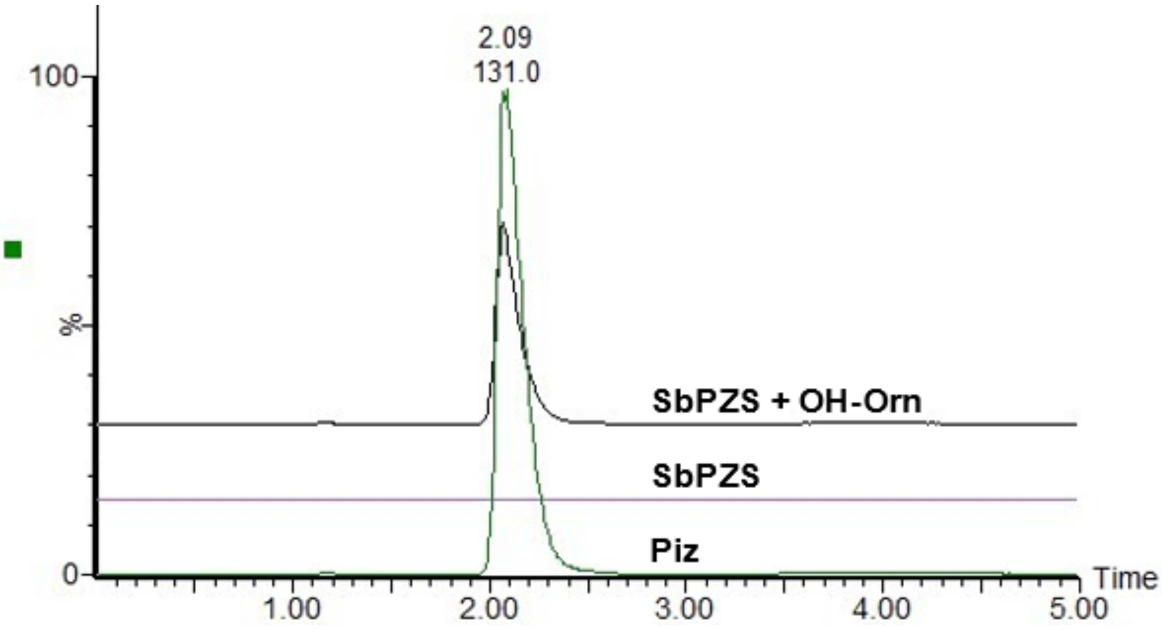

Figure S4: LC-MS analysis of SbPZS-catalyzed formation of L-piperazic acid. Extracted ion chromatograms (SIR, m/z 131.0 [M+H]+) are shown for the reaction containing heme-reconstituted SbPZS and OH-Orn (top), the no-substrate control (middle), and an authentic L-piperazic acid standard (bottom).

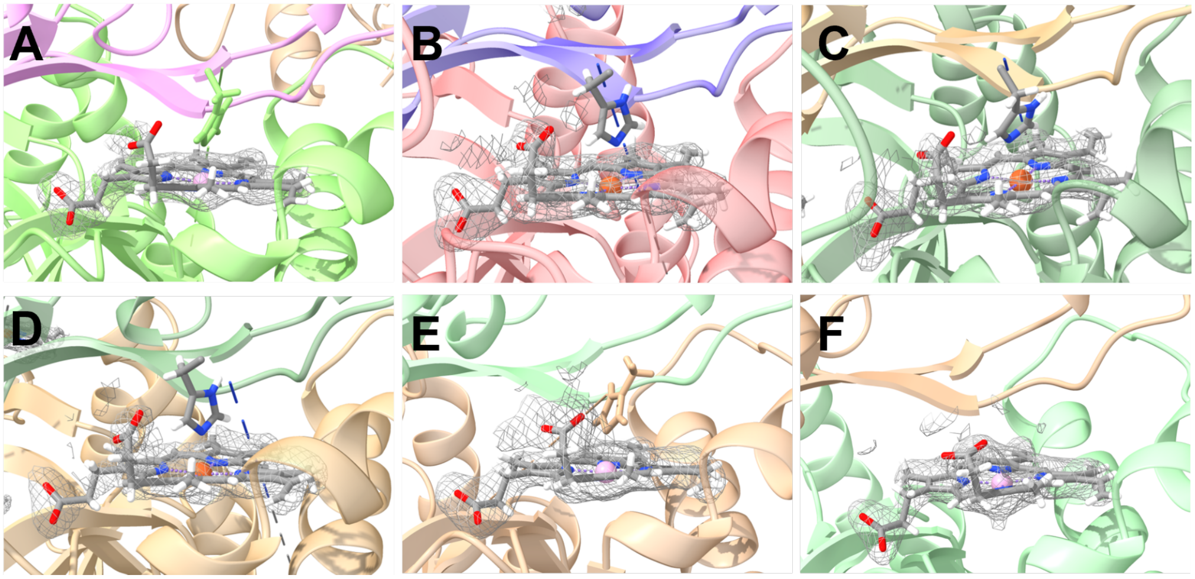

Figure S5: Polder omit maps (contoured at 3σ) for the heme cofactor in chains A-F of holo-SbPZS, calculated after removal of the heme and exclusion of bulk solvent from the active site.

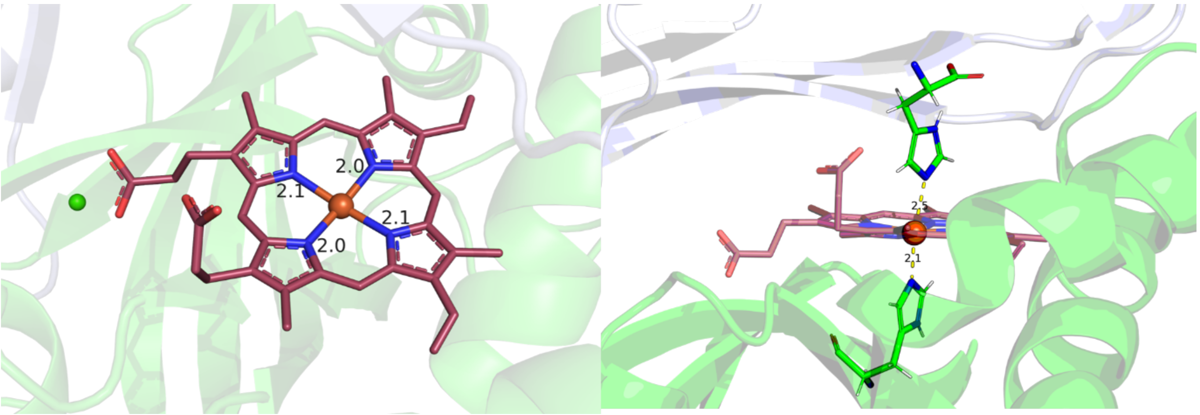

Figure S6: SbPZS active site showing the distances of the metal coordination bonds between Fe and His-61 and between Fe and the four nitrogen in heme.

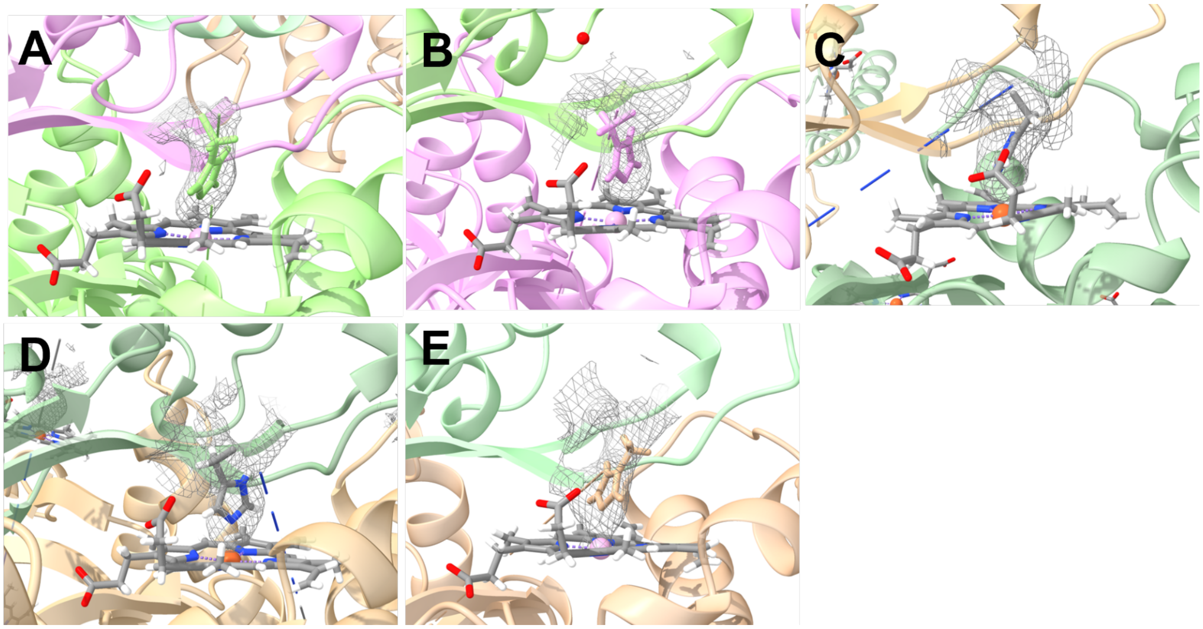

Figure S7: Polder omit maps (contoured at 3σ) for the additional histidine-like density above the heme in chains A-E. The density is consistent with a C-terminal His-tag residue, but no continuous density links it to a specific protein chain. No corresponding density was observed in chain F.

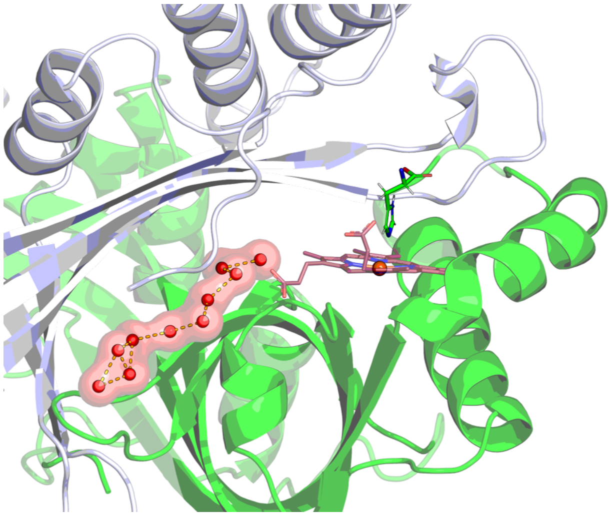

Figure S8: Water-filled tunnel at the SbPZS dimer interface, leading toward the active site. The two protein chains are shown in blue and green, ordered water molecules as red spheres, and hydrogen bonds between water molecules are indicated by dashed lines.

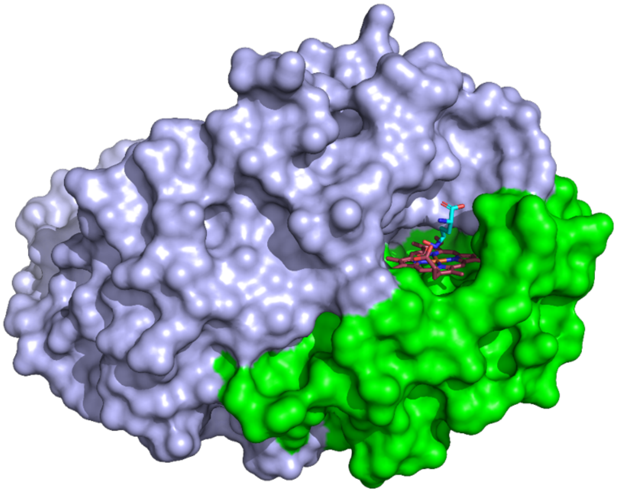

Figure S9: Surface representation of the SbPZS dimer with heme and modeled N5-hydroxy-L-ornithine in the active site. The two protein chains are colored blue and green.

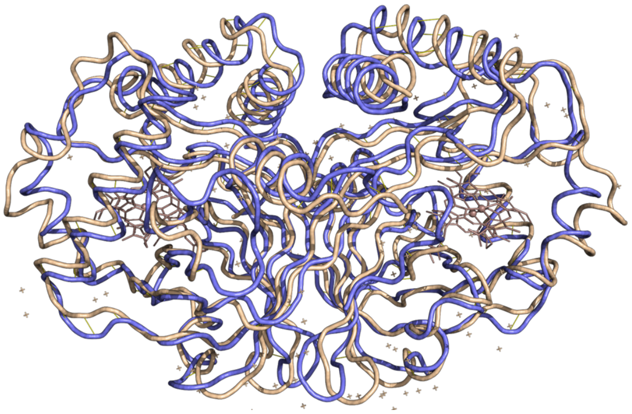

Figure S10: Structural alignment of SbPZS (PDB: 9QLK, blue) and heme-bound PaiB (PDB: 9VYC, wheat) with a Cα RMSD of 1.6 Å over 295 superimposed Cα atoms.

Protein substrate dynamics:

Replicate 1

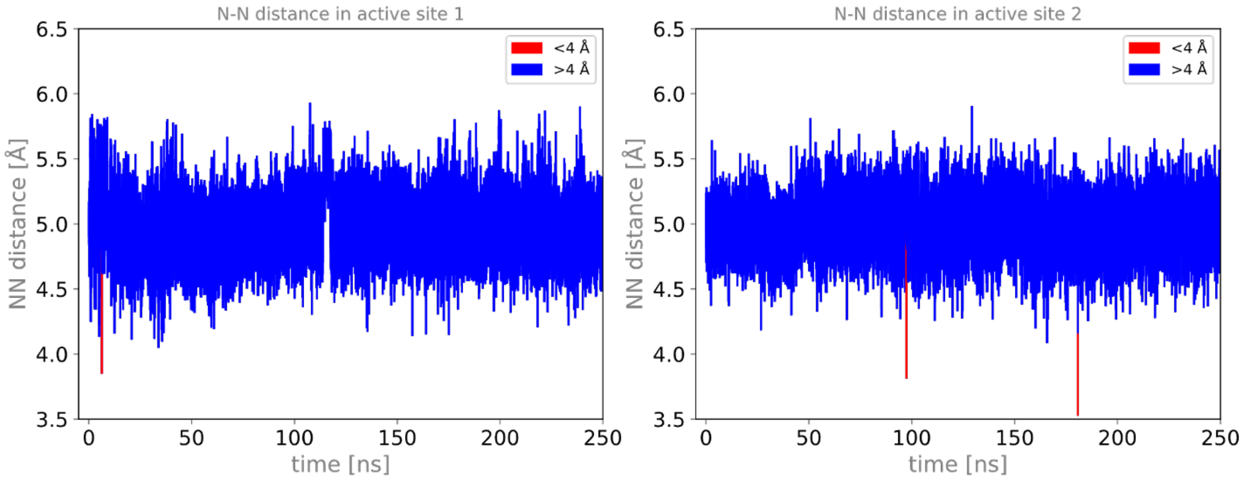

Replicate 2

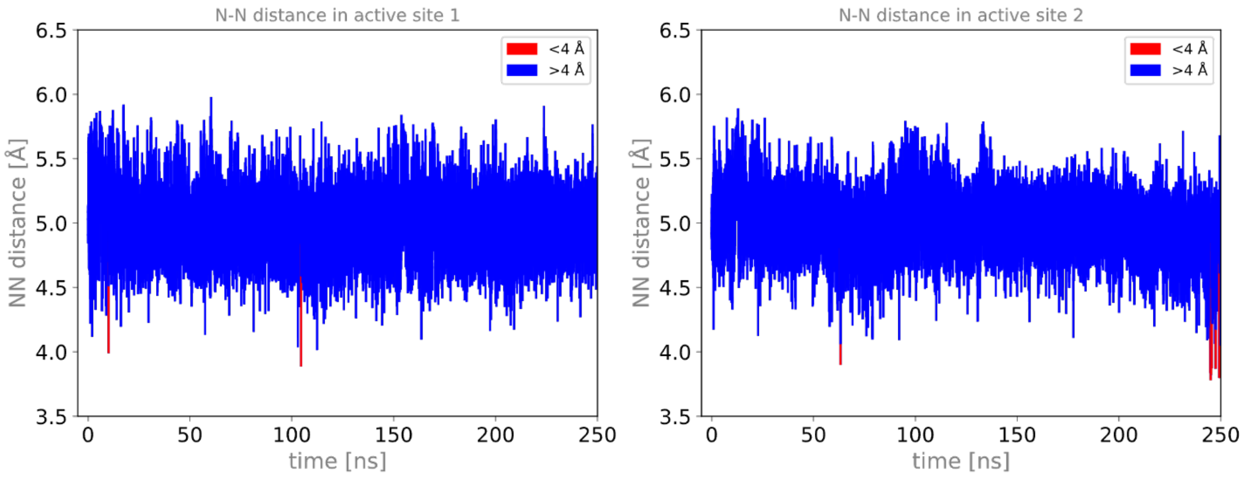

Replicate 3

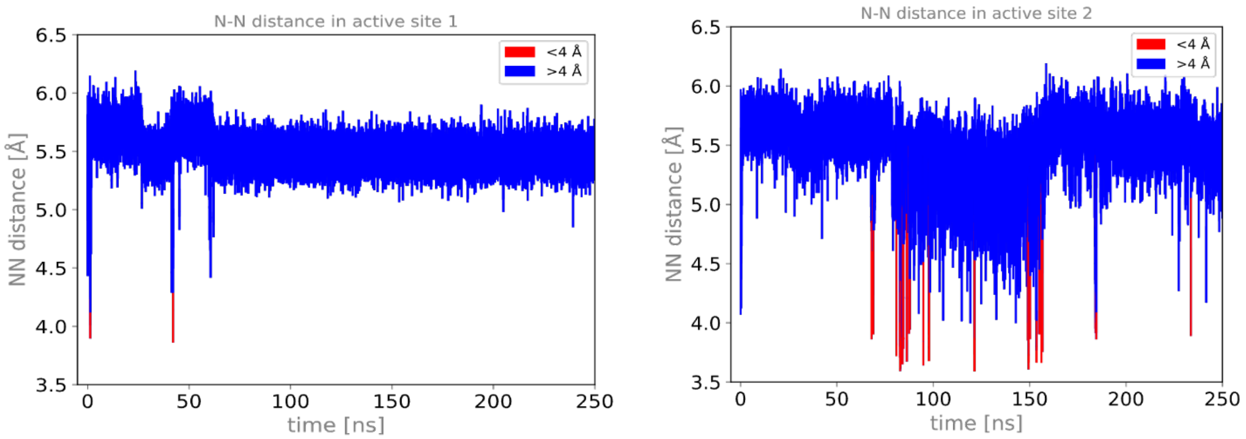

Figure S11: Intramolecular N-N distance in N5-hydroxy-L-ornithine across three independent 250 ns MD simulations of the SbPZS-heme-substrate model. Distances below 4 Å were used as a descriptor of closed substrate conformations.

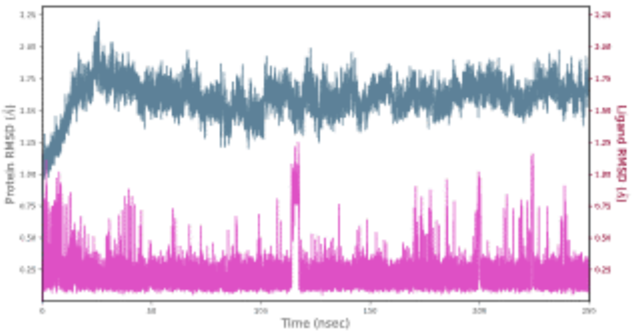

Figure S12: Protein backbone and ligand RMSDs during the MD simulations of the SbPZS-heme-substrate model.

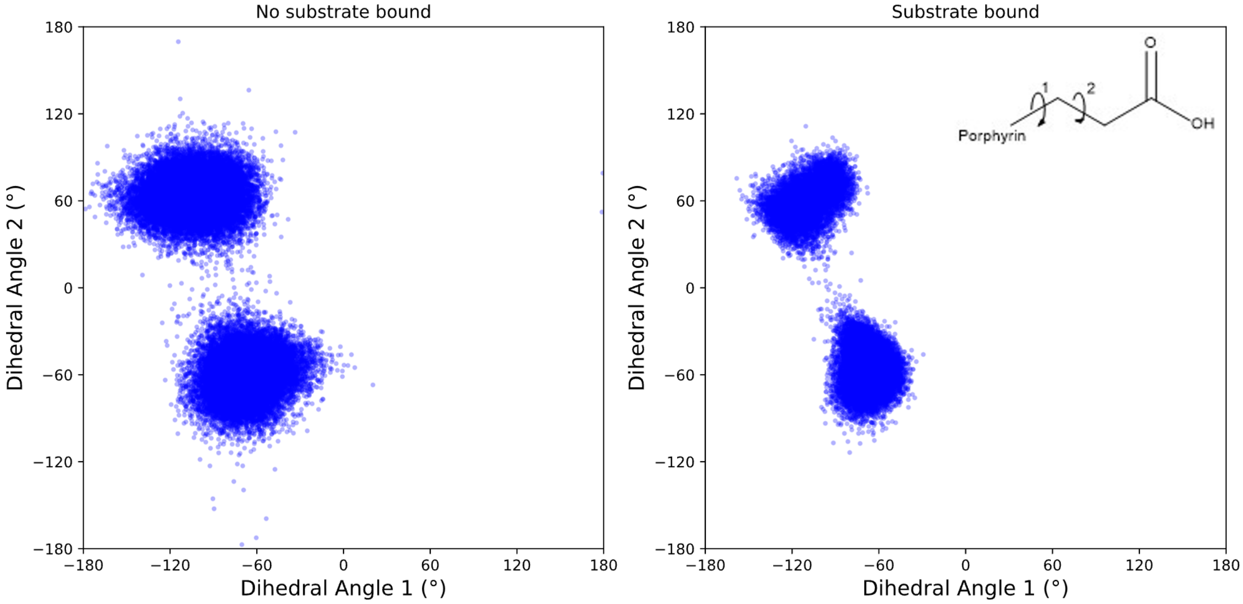

Figure S13: Conformational sampling of the heme propionate side chain, monitored by two dihedral angles during MD simulations with and without substrate. Each point represents a trajectory snapshot.

3. Supplementary Tables

**Protein**	**SbPZS**
**Wavelength**	0.8731 Å
**Resolution range**	51.79 - 2.14 (2.17 - 2.14) Å
**Space group**	P 3_2
**Unit cell**	a=b=141.708 Å, c=57.128 Å, α=β=90°, γ=120°
**Total reflections**	415749 (16975)
**Unique reflections**	78184 (3163)
**Multiplicity**	5.3 (5.4)
**Completeness (%)**	99.95 (100.00)
**Mean I/sigma(I)**	7.00 (0.90)
**Wilson B-factor**	36.01
**R-merge**	0.1437 (1.288)
**R-meas**	0.1596 (1.428)
**R-pim**	0.069 (0.6121)
**CC1/2**	0.996 (0.382)
**CC***	0.999 (0.743)
**Reflections used in refinement**	70738 (2822)
**Reflections used for R-free**	3541 (156)
**R-work**	0.1926 (0.2784)
**R-free**	0.2651 (0.3086)
**Number of non-hydrogen atoms**	10656
macromolecules	10020
ligands	298
solvent	338
**Protein residues**	1274
**RMSD (bonds)**	0.009 Å
**RMSD (angles)**	1°

**Ramachandran favored (%)**	95.15
**Ramachandran allowed (%)**	4.53
**Ramachandran outliers (%)**	0.32
**Rotamer outliers (%)**	3.16
**Clashscore**	3.94
**Average B-factor**	47.34
macromolecules	47.8
ligands	40.78
solvent	39.41

Table S1: Data collection and refinement statistics of the SbPZS crystal structure

PZS structures' comparison:

**Reference**	**Mobile**	**RMSD**	**Aligned C-alpha atoms**
PZS_dimer1	PZS_dimer2	0.168	355
PZS_dimer1	PZS_dimer3	0.151	359
PZS_dimer1	9VYC	1.536	293
PZS_dimer1	9JN5_dimer1	0.614	343
PZS_dimer1	9JN5_dimer2	0.575	338
PZS_dimer1	9EBM	0.619	346
PZS_dimer1	2OL5	1.619	279
PZS_dimer1	9KEA_dimer2	0.826	352
PZS_dimer1	9KEA_dimer1	0.810	353
PZS_dimer2	PZS_dimer3	0.142	365
PZS_dimer2	9VYC	1.604	295
PZS_dimer2	9JN5_dimer1	0.730	356
PZS_dimer2	9JN5_dimer2	0.649	342
PZS_dimer2	9EBM	0.585	346
PZS_dimer2	2OL5	1.680	280
PZS_dimer2	9KEA_dimer2	0.819	352
PZS_dimer2	9KEA_dimer1	0.781	350
PZS_dimer3	9VYC	1.594	294

PZS_dimer3	9JN5_dimer1	0.697	351
PZS_dimer3	9JN5_dimer2	0.633	340
PZS_dimer3	9EBM	0.591	343
PZS_dimer3	2OL5	1.673	279
PZS_dimer3	9KEA_dimer2	0.777	353
PZS_dimer3	9KEA_dimer1	0.767	350
9VYC	9JN5_dimer1	1.533	235
9VYC	9JN5_dimer2	1.545	236
9VYC	9EBM	1.541	333
9VYC	2OL5	0.344	321
9VYC	9KEA_dimer2	1.877	343
9VYC	9KEA_dimer1	1.793	330
9JN5_dimer1	9JN5_dimer2	0.124	401
9JN5_dimer1	9EBM	0.825	373
9JN5_dimer1	2OL5	1.518	222
9JN5_dimer1	9KEA_dimer2	0.914	359
9JN5_dimer1	9KEA_dimer1	0.856	361
9JN5_dimer2	9EBM	0.789	369
9JN5_dimer2	2OL5	1.531	223
9JN5_dimer2	9KEA_dimer2	0.896	352
9JN5_dimer2	9KEA_dimer1	0.886	363
9EBM	2OL5	1.659	318
9EBM	9KEA_dimer2	0.898	363
9EBM	9KEA_dimer1	0.855	362
2OL5	9KEA_dimer2	2.007	325
2OL5	9KEA_dimer1	1.924	315
9KEA_dimer2	9KEA_dimer1	0.136	334

Table S2: Pairwise structural comparison of SbPZS (9QLK), PipS (9EBM), KtzT (9JN5), LnzB (9KEA), heme-bound PaiB (9VYC), and PaiB (2OL5). Superpositions and RMSD values were calculated using the program PyMOL (Schrödinger Inc.).
